# Cell Reversal From a Differentiated to a Stem-Like State at Cancer Initiation

**DOI:** 10.3389/fonc.2020.00541

**Published:** 2020-04-15

**Authors:** João Carvalho

**Affiliations:** CFisUC, Department of Physics, University of Coimbra, Coimbra, Portugal

**Keywords:** carcinogenesis, epigenetics, cancer stem cells, dedifferentiation, reprogramming, cancer

## Abstract

Even if the Somatic Mutation Theory of carcinogenesis explains many of the relevant experimental results in tumor origin and development, there are frequent events that are not justified, or are even contradictory to this widely accepted theory. A Cell Reversal Theory is presented, putting forward the hypothesis that cancer is originated by reversal of a differentiated cell into a non-differentiated stem-like state, by a change of its intrinsic epigenetic state, following a perturbation on the cell and/or its microenvironment. In the current proposal a cluster of cancer stem cells can be established, without the strict control mechanisms of a normal stem cell niche, and initiate a tumor. It is proposed that a reversal to a pluripotent state is at tumor origin and not tumor progress that prompts cell dedifferentiation. The uncontrolled proliferation of cancer stem cells causes a microenvironment disorganization, resulting in stressful conditions, like hypoxia and nutrient deprivation, which induces the genetic instability characteristic of a tumor; thus, in most cases, mutations are a consequence and not the direct cause of a tumor. It is also proposed that metastases result from dedifferentiation signaling dispersion instead of cell migration. However, conceivably, once the microenvironment is normalized, the stem cell-like state can differentiate back to a mature cell state and loose its oncogenic capacity. Therefore, this can be a reversible condition, suggesting important therapeutic opportunities.

## Introduction

The Somatic Mutation Theory (SMT) of carcinogenesis (1) explains cancer origin by an accumulation of genetic mutations on tumor suppressor genes and on oncogenes that are transmitted to its lineage. It follows that the hallmarks of cancer (2) derive from successive mutations producing advantageous biological capabilities, in a multistep process of tumor development. This widely accepted theory explains many cancer features, from hereditary cancers to successful therapies targeting the product of mutant genes (1). But there are also many important events that are contradictory to its predictions and some *ad-hoc* modifications must be introduced to explain them, leading to serious inconsistencies. There are many reports of zero mutations found in some tumors (3), whereas malignant properties are a result of changes in the DNA methylation pattern and not in its sequence (1). Additionally, there are a few non-genotoxic carcinogens, like chloroform and p-dichlorobenzene (4), which induce cancer without direct modifications to DNA. There are experiments where mutated genes are introduced into animals' cells and lead to cancer onset (5, 6), but this outcome can be due to the procedure burden, causing a transition of the cell from a “normal” to an “abnormal” epigenetic state.

Cell genes on DNA can be considered as a large collection of software routines, each one of them with instructions to produce a particular protein. All cells have the same collection of these routines, and they are almost the same in every human being. But, according to its tissue of origin, cells shape, behavior and fate are very different and can change considerably during development. The particular cell epigenetic state (7) is fundamental as it defines the correct course to run the program in that particular circumstances, defining the number and order of calls of different genes, and thus their transcription and protein production rates. This is executed in such a way that the cell survives and performs its duty in the appropriate fashion for the human being continuity and development. Each particular differentiated cell type corresponds to a distinct epigenetic state, specified by, for instance, DNA methylation and histone modification (8, 9), which depends on the tissue where it is placed (its microenvironment) and the development stage of the individual (the particular moment in its maturation history).

**Figure 1:**
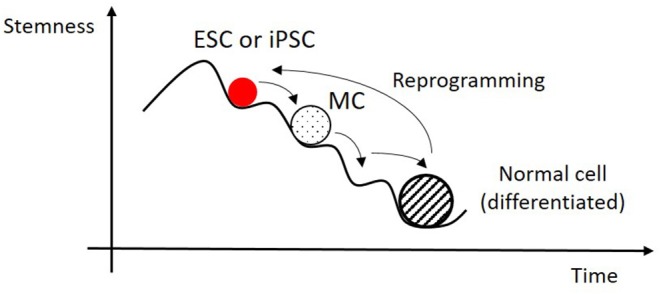
In a highly simplified projection of a very complex epigenetic landscape, an embryonic stem cell (ESC) or an induced pluripotent stem cell (iPSC) can differentiate by successive steps between locally stable states until it reaches a fully differentiated mature state. Time reversal from this state to a pluripotent one is possible, in special conditions, by cell reprogramming. This can take place in a single or a multistep course, which can include Multipotent Cell (MC) states.

A viable cell is then a point of stability on the epigenetic (very large) n-dimensional landscape of possible gene transcription rates and active signaling pathways (see Figure 1). It is in one of a multitude of possible phenotypes, where it can be, for instance, a muscle, a bone or an endothelial cell. All of them have the same genetic code, inherited from the same zygote cell, but they have very different gene expression patterns. These epigenetic states are time and location specific, and their modification defines a new state which can be or not stable. If it is not on a viable program the cell will die and not reproduce.

In an adult, stem cells are present in niches, which are regions on a tissue with a very specific microenvironment. These cells interact with each other and with the surrounding more differentiated cells in order to renew cell population, in a highly controlled fashion, by proliferation and differentiation (10).

### Cell Reversal Theory: Stem-Like Cells Due to Epigenetic Reversal of Mature Cells at Tumor Origin

A hypothesis for carcinogenesis, the Cell Reversal Theory (CRT), states that due to a perturbation (a potential carcinogenic event) on the cell and/or on its environment, the cell does a transition to a different epigenetic state which, due to the absence of adequate control mechanisms at its current time/place, can lead, in special circumstances, to abnormal proliferation. A cell can enter on the wrong epigenetic program, according to its environment and stage of development, and become what is labeled as a cancer stem cell. It is thus suggested that excessive proliferation rate is due to the absence of the right control mechanisms from the environment that would constrain its behavior and not (only) a result of genetic mutations, as assumed by SMT. Initial under or overexpression of particular genes is then, in many tumors, due to epigenetic factors and not to genetic mutations. Reversal of a differentiated cell into a stem cell-like status, in an environment very different from the stem cell niche, tightly regulated by genetic and epigenetic factors (11), can lead to a chaotic and uncontrolled proliferation. In a multitude of possible cell epigenetic states, only a very small fraction is viable and has survival advantages. So it should be much more probable, and efficient, for a cancer cell to run a program that was evolutionarily selected and optimized, the stem cell or pluripotent program, than by the successive acquisition of all the right characteristics and capacities for enhanced proliferation, cell-death resistance and invasion.

**Figure 2:**
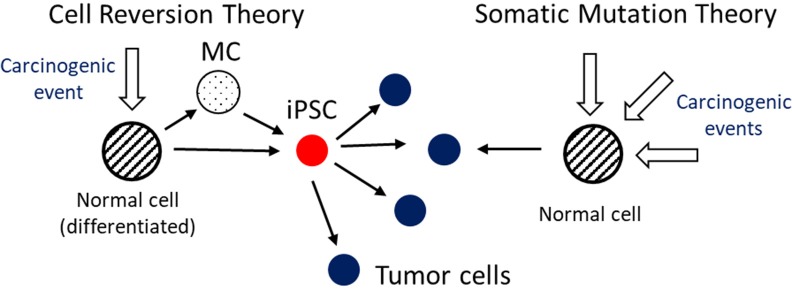
In the Cell Reversion Theory a normal (differentiated) cell can complete a transition to an induced pluripotent stem cell (iPSC) state, of more stem cell like nature, in one or more steps [eventually passing through Multipotent Cell (MC) states], due to a perturbation (e.g., chemical or mechanical) to its conditions and/or its environment equilibrium. The iPSC can proliferate, and differentiate, in an unrestrained way generating a stressful cell environment, like hypoxia, a mutagenic condition. This cell, according to the presented hypothesis, can be at tumor origin. In the Somatic Mutation Theory successive mutations by carcinogenic events lead to the tumor phenotype.

In an earlier stage of organism development (12), as embryonic stem cells (ESC), or later as induced-pluripotent stem cells (iPSC), cells present a higher proliferation rate than at a mature state. In the event of a later cell reversal to one of these states, the cell doesn't receive the right chemical and mechanical signals from the microenvironment in which it is situated to control its development, and this can result in an uncontrollable multiplication. This is one of the risks found on reprogramming techniques being developed for regenerative medicine: iPSC, and also ESC, show a high carcinogenic capacity and must switch to a differentiated state before transplantation into the new tissue (13) (see Figure 2). Human embryonic stem (hES) cells conduce to teratoma formation, probably due to expression of survivin upon differentiation (14). However, a cell doesn't need to go all the way to the ESC or iPSC state, can make a transition to an intermediate multipotent state with increased capability of proliferation.

This hypothesis is different from the atavism theory (15), which proposes a cell de-evolution into a more primitive form of life. A cell running the “wrong” epigenetic program for its place/time would, in most of the cases, die, as its state is not adequate for survival in these particular conditions. But, in some special circumstances, could survive and thrive, being at the origin of a tumor. The present proposal is also different from the Tissue Organization Field Theory (TOFT) (16) in the sense that no special morphostat substance is necessary to exist and to be perturbed in order to initiate a tumor. But, as in TOFT, carcinogenesis can also have origin on a perturbation of the tissue environment, leading to a transition between epigenetic states, from normal to pluripotent. The review article by Friedmann-Morvinski and Verma (17) presents a theory with similarities to CRT for the origin of Cancer Stem Cells (CSC) but proposes them as a consequence of tumor progression and not at its origin. They point to the correspondence between the mechanisms of cells reprogramming into a pluripotent state and the dedifferentiation of tumor cells to CSC by epigenetic resetting. There are several theories about the origin of CSC, reviewed in Nimmakayalaa et al. (18), including cell fusion, horizontal gene transfer, mutations, metabolic reprogramming and dedifferentiation of non-CSC into CSC (in response to stress, wounding or hypoxia, as the hypothesis proposed here). CSC are an intensely researched subject, their existence being gradually accepted for many cancers. Some good reviews on the topic [as (19, 20)] examine and discuss the different hypothesis associated with the acquisition of stemness and tumor heterogeneity, including the effect of epigenetics, microenvironment and mutations.

It was shown that genes used on cells' reprogramming, like the Oct3/4, Sox2, Klf4 and c-Myc (the OSKM cocktail) (21), are also linked to tumors. As shown in Vaux (1), some experiments use oncogenes to activate iPSC; ESC genes and networks, like Oct3/4, SOX2 and Nanog, are activated on cancer initiation and progress (18). This can be interpreted as an association between cells pluripotency, after their regression to a more stem-cell like state, and carcinogenesis (12). Stem and cancer cells' phenotypes share some similarities, the two being in a proliferative state, are invasive and can be considered potentially immortal (22). Also they both show self-renewal capability and block differentiation (22); they are primitive and undifferentiated (1, 23). Our hypothesis is then that carcinogenesis can be due to resurrection of an early stem cell-like behavior, with expression of stem cell transcription factors, in an inappropriate location and time (22).

Probably some cancer cells maintain their stemness competence (24) and these can move into another place and start a new tumoral colony (a metastasis). Or, as a new hypothesis, which seems much simpler and probable, metastases are due to dedifferentiation signaling dispersal and not a result of cell migration. If the epigenetic state changing molecules reach a tissue with susceptibility for cell dedifferentiation, due to stress or some perturbation event on its microenvironment, it is possible to reproduce the transition event to CSC, triggering a tumoral initiation event at a different place.

In the mobilization therapy for bone marrow transplantation (25), stem cell like pluripotent cells are forced into the blood stream from the bone marrow of healthy human donors before being transplanted into a patient. According to the CRT hypothesis this could introduce an increased risk of cancer development, which was not found (26). Possible explanations are the difficulty of stem cells to extravasate the capillary vessels in their relatively short circulation time, or the just proposed hypothesis that dedifferentiation signaling diffusion is involved in metastasis and not cell migration as it is usually considered.

### Relevance of the Microenvironment Perturbation for State Transition

The cause of epigenetic program change can be a perturbation to the cell and/or to its environment, a carcinogenic event, which disturbs the equilibrium conditions beyond what the cell can recover from, and eventually moves it toward another stable and viable point on the epigenetic landscape [for instance, by methylation/demethylation processes, (8)]. The stress event (for instance, caused by exposure to a chemical or to radiation) can overwhelm the cell control and feedback systems and make the cell change its epigenetic program as it tries to respond to the disruptive incident. As it endeavors to adapt and survive in new conditions, it can revert its differentiation status. The disorganized microenvironment then becomes a cradle of cells on different differentiation stages and epigenetic states, including pluripotent CSC.

In this scenario, tumoral genetic mutations are, in most cases, a cancer symptom and not its cause, as it was shown in many clinical examples (27). The genetic instability accompanying the excessive cell proliferation and the epigenetic changes (8) can be, at least partially, at the origin of the high mutation rate found in tumors. The uncontrolled cell number expansion results in a hostile microenvironment (hypoxia, nutrient depletion, low pH) that induces mutagenesis, DNA damage and impairment of DNA repair (28, 29). This is shown by the indication that tumors are, in many cases, not a clonal grouping of cells but polyclonal, due to this genome mutability (8), and, in general, tumor tissue exhibits large heterogeneity on its differentiation status. Even hereditary cancers show some paradoxical behaviors, where, for instance, Xeroderma pigmentosum patients (30), a genetic disease characterized by defects on the DNA repair mechanisms in all cells, show a high rate of skin cancer but not of other cancers, as would be expected. In another example, mutated genes inserted into animals can lead to cancer, but in some cases driver (cancer originator) mutations are not present in the resulting tumor (5, 31). In CRT hereditary cancers can be explained by transmitted variability that make cells more prone to transition to the undifferentiated state at tumor origin. It also explains why cancer is more probable in old age as abnormal cell methylation can be an ordinary result of aging (32), which can make them more susceptible to epigenetic transition (this can be due to an impairment on the activation of genes involved in cell differentiation) (32). There are clear evidences that overweight and obesity are linked to an increased risk of some types of cancer. This outcome can be explained by cellular environment disorganization due to metabolic and inflammatory modifications in adipose tissue, which disrupts homeostasis (33) and promotes epigenetic transition.

In transplantation experiments (34) it was shown that exposure of tissue stroma to a carcinogen (N-nitrosomethylurea) is at the origin of a tumor in the epithelial layer, when placed in contact with the treated stroma, independently of the epithelial cells being or not exposed to the carcinogen. The same procedure applied on the epithelial layer would not lead to a tumor if the stroma was not also treated with the carcinogen. This result can be interpreted by a disturbance of the normal signaling and/or cell state equilibrium from the exposure of stroma to the chemical substance, stimulating the production of an epigenetic state changing molecular cocktail, which induces the transition to pluripotency. It was also found that, in some cases, transplant of tumor cells into a normal tissue leads to their reversion to normal state, which can be explained by their differentiation on the new environment.

The hypothesis that the acquisition of stem-like capabilities by differentiated cell reprogramming, induced by a cell and/or tissue perturbation, can lead to its tumorigenic behavior, including induction of genomic instability and consequent mutations from microenvironment adverse conditions, can then interpret contradictory findings not explained, in a straightforward way, by SMT. Other hypothesis doesn't seem reasonable, where the stem cell like capability is a consequence of tumor progress (17) and CSC origin from cancer cells and not directly from normal mature cells.

Some relevant experimental results discussed in this work favor the CRT model of carcinogenesis, as it justifies many of the results contradictory to SMT predictions while giving a plausible explanation to tumor origin. But, much probably, one or the other tumorigenic events are present in different cancers, and may even cooperate in some circumstances. For instance, this can happen when a cell mutation occurs that leads to a deregulation of its epigenetic control mechanisms and this promotes its transition to a stem-like state. Or the other way around, when the perturbation of the tissue microenvironment created by the uncontrolled stem cell proliferation produces the right conditions for the genetic instability common in tumors. Then, from the initial stem cell properties it can evolve into the mutated and differentiated states present in a tumor.

### Proposed Tests of the Carcinogenesis Hypothesis

Several tests can be performed, in different tissues, to assess the current hypothesis, in particular for solid tumors. A particular effort should be placed in the search for stem cell markers in the initial tumor stages, originated by mutagenic and non-mutagenic processes. The evolution of these markers, as the tumor grows in diverse organs and for specific cancer types, would produce relevant evidence for this hypothesis examination. Stress experiments, from hypoxia events to introduction of foreign bodies and cells on a tissue, or addition of external chemical substances, can be used to prove the CRT hypothesis, which doesn't involve mutagenic episodes at the tumor origin. The work by Nakada et al. (35) describes the regeneration of cardiomyocytes by a special procedure involving deep hypoxia. These results can be interpreted as resulting from hypoxia stress inducing a transition of some cardiomyocytes to a stem cell like state, recovering the lost neonatal myocardium regenerating capacity. Similar experiments can be conceived to test for epigenetic transitions by stress events contributing to tumor initiation. It was shown that tumor cells with mutated or down-regulated BRCA1, a multifunctional protein involved in epigenetic control and DNA repair, present an increased expression of CSC-associated markers CD44 and ALDH1A (11). It was found (11) that down-regulation (reconstitution) of BRCA1 resulted in significant increase (decrease) of CSC-like populations in breast cancer. This seems to confirm that genes involved in cancer are also associated with cell reprogramming, and other cancer related genes can be tested for analogous results. Another possible experiment to prove the hypothesis is to test the effect of transplant of stem cells into an adult animal and to check if these cells, when introduced at the wrong time of animal development, can be at the origin of a tumor. This was already proven to occur with iPSC and ESC (13, 36) but the different steps of tumorigenic progress can be more precisely characterized, in particular the transition and/or evolution of the cell epigenetic program. Progression on the level of tumor stemness (37) can also be evaluated to check how the cell differentiation state changes with tumor expansion. Recent stemness measurements (37), applying a machine learning approach to define different indices (involving, for instance, mRNA expression, histone markers and DNA methylation), have shown that stemness is lower in normal cells, larger in primary tumors and highest in metastasis. These results can be related to cell reprogramming into a pluripotent state (with stem cell-like properties), in a dedifferentiation process, as being at the tumor origin, followed by successive divisions and cells gradual differentiation. Stemness is then lower at an earlier primary tumor than at a later onset metastasis.

## Conclusions and Outlook

If this hypothesis is proved right, a potential therapeutic approach is to pursue the normalization of the tumor environment, according to tissue type, and force cells back to the right epigenetic program. This can be achieved by inducing their differentiation, corresponding to the normal state at that particular development stage and location. There are reported cases of tumors' remission by inhibition of enzymes (5, 38), the ones specifically activated by the mutated genes. This result can also be explained by a transition of cells back to a differentiated state through a change on the biochemical environment and/or on the active signaling pathways. Several examples of spontaneous regression of tumors were reported (5, 39), where tumor cells revert to normal tissue. Tissue normalization implies an adequate supply of oxygen, with the presence of a suitable vasculature network and blood flow, and access to nutrients and the requisite chemical signals. Probably the addition of a specific cocktail of transcription factors will be needed to prompt a state transition to normalcy, or by tuning the level of epigenetic regulating enzymes. A possible approach to cancer remission would then involve differentiation therapies (37). This procedure will not eliminate cells that already suffered mutations but eventually can differentiate the CSC and thus eliminate this particular niche, which is a probable cause of cancer relapse and metastases. This therapy can also be used to prevent cancer and/or to decrease cancer risk. A recent paper (40) reviews possible therapeutic strategies against CSC. Induced differentiation therapies are being considered and a treatment with retinoid acid was proposed (41, 42), which is also under examination to be used in cancer prevention. Another organic molecules are being examined, as vitamin D_3_ (43), and they can be used as cancer inhibitors and/or as an adjuvant cancer therapy to reduce or defeat the CSC niche. A different strategy is the search and use of embryonic antigens, as A19, which was demonstrated (44) to be an effective targeted agent for Erbb-2 expressing cancers. Cell surface antigens are widely used to characterize embryonic stem cells, in particular to monitor their differentiation (45), and such antigens, which include both glycolipids and glycoproteins, can also be exploited for cancer diagnosis and therapy. If this carcinogenesis model is correct, in the sense that it explains, at least partially, the events that lead to cancer initiation by dedifferentiation of mature to stem cells, it can also be used in the opposite way. Known carcinogenic events can be used to reprogram cells to a pluripotent state and it can be compared with other techniques in terms of efficiency, simplicity and safety.

From the evolutionary point of view, cancer is greatly deleterious for the individual survival and proliferation, and has been highly suppressed during life evolution. Cells possess many redundant protection mechanisms to avoid cancer, from DNA error check and correction to apoptosis, so it is very hard to get rid of all of them and still be viable and have a competitive advantage with respect to normal cells. But it is not possible to suppress the mechanism of epigenetic reversion to an earlier development (pluripotent) state as this state is a fundamental step on a living being maturation. Then the (potentially dangerous) pluripotent state is so important in embryogenesis and tissue homeostasis that it cannot be eliminated by evolution, even if it can later be at a tumors' origin. Therefore, the reversal of a normal mature cell into a stem-like cell state can explain many tumor initiation and progression observations found to be contradictory with SMT and open new therapeutic avenues. Anyway, one hypothesis can be at the origin of some tumors and the other one for separate cases, but it is also possible that both are present and cooperating in cancer initiation.

## Author Contributions

The author confirms being the sole contributor of this work and has approved it for publication.

### Conflict of Interest

The author declares that the research was conducted in the absence of any commercial or financial relationships that could be construed as a potential conflict of interest.
